# Specialization for resistance in wild host-pathogen interaction networks

**DOI:** 10.3389/fpls.2015.00761

**Published:** 2015-09-23

**Authors:** Luke G. Barrett, Francisco Encinas-Viso, Jeremy J. Burdon, Peter H. Thrall

**Affiliations:** ^1^Commonwealth Scientific and Industrial Research Organization Agriculture FlagshipCanberra, ACT, Australia; ^2^Centre for Australian National Biodiversity Research, Commonwealth Scientific and Industrial Research OrganizationCanberra, ACT, Australia

**Keywords:** rust, virulence, avirulence, specialist, generalist, bipartite, temporal, spatial

## Abstract

Properties encompassed by host-pathogen interaction networks have potential to give valuable insight into the evolution of specialization and coevolutionary dynamics in host-pathogen interactions. However, network approaches have been rarely utilized in previous studies of host and pathogen phenotypic variation. Here we applied quantitative analyses to eight networks derived from spatially and temporally segregated host (*Linum marginale*) and pathogen (*Melampsora lini*) populations. First, we found that resistance strategies are highly variable within and among networks, corresponding to a spectrum of specialist and generalist resistance types being maintained within all networks. At the individual level, specialization was strongly linked to partial resistance, such that partial resistance was effective against a greater number of pathogens compared to full resistance. Second, we found that all networks were significantly nested. There was little support for the hypothesis that temporal evolutionary dynamics may lead to the development of nestedness in host-pathogen infection networks. Rather, the common patterns observed in terms of nestedness suggests a universal driver (or multiple drivers) that may be independent of spatial and temporal structure. Third, we found that resistance networks were significantly modular in two spatial networks, clearly reflecting spatial and ecological structure within one of the networks. We conclude that (1) overall patterns of specialization in the networks we studied mirror evolutionary trade-offs with the strength of resistance; (2) that specific network architecture can emerge under different evolutionary scenarios; and (3) network approaches offer great utility as a tool for probing the evolutionary and ecological genetics of host-pathogen interactions.

## Introduction

Interactions between wild hosts and their pathogens are typically characterized by high levels of genetic diversity for partner specificity, such that pathogens vary in their capacity to infect individual hosts, and hosts are likewise variable in their ability to resist attack by individual pathogens (Laine et al., 2011; Tack et al., 2012). Furthermore, hosts and pathogens vary not only in terms of specificity for individual partners, but also in terms of partner range (i.e., breadth of resistance; pathogen host-range) (Barrett et al., 2009a; Barrett and Heil, 2012). Understanding the factors that influence the genetic specificity of host-parasite interactions is important as patterns of association underlie susceptibility to disease, and thus many aspects of disease dynamics and epidemiology (see Thrall et al. this issue for review). However, the ecological and evolutionary factors that determine variation in individual specialization and partner breadth in host-pathogen interactions are generally not well understood.

Polymorphisms for resistance and infectivity in wild host–pathogen interactions are typically measured by performing pair-wise infections of host lines by pathogens isolated from natural populations. The results of such pair-wise infections can be represented as a matrix or bipartite network (see Box 1 for description), where the rows indicate host genotypes, the columns indicate pathogen genotypes, and the cells within the matrix indicate whether a given combination results in resistance or infectivity. Bipartite-network analytical methods and approaches have been used extensively in observational studies of mutualistic (e.g., Vázquez et al., 2009; Guimarães et al., 2011), and host-parasite (e.g., Vázquez et al., 2005; Morris et al., 2014) networks, but have been only rarely used to analyse patterns of phenotypic variation in host resistance and pathogen infectivity (but see Flores et al., 2012; Poisot et al., 2013). Network approaches are of potential utility for analysing host-pathogen interactions because the statistical structure of such networks offers a standardized framework for describing and quantifying patterns of specialization within host-pathogen interactions (Blüthgen et al., 2006; Vacher et al., 2008; Weitz et al., 2013). Specificity can be simply estimated for individuals, or the network as a whole, based on the number of links (i.e., resistance or infectivity interactions) an individual has with its antagonistic partners (Poisot et al., 2013). In addition, patterns of specialization can be characterized by estimating two key network properties, nestedness and modularity, within the infection or resistance matrix. Nestedness is a network property describing the extent to which specialists interact with a subset of partners that also interact with generalists, whereas modularity indicates the extent to which resistance or infectivity interactions can be partitioned into distinct groups, each of which has many internal interactions but few with other groups (see Box 1 for a detailed description of these key statistical properties).

Box 1 **The structure of host-pathogen coevolutionary networks.**

**Figure 1:**
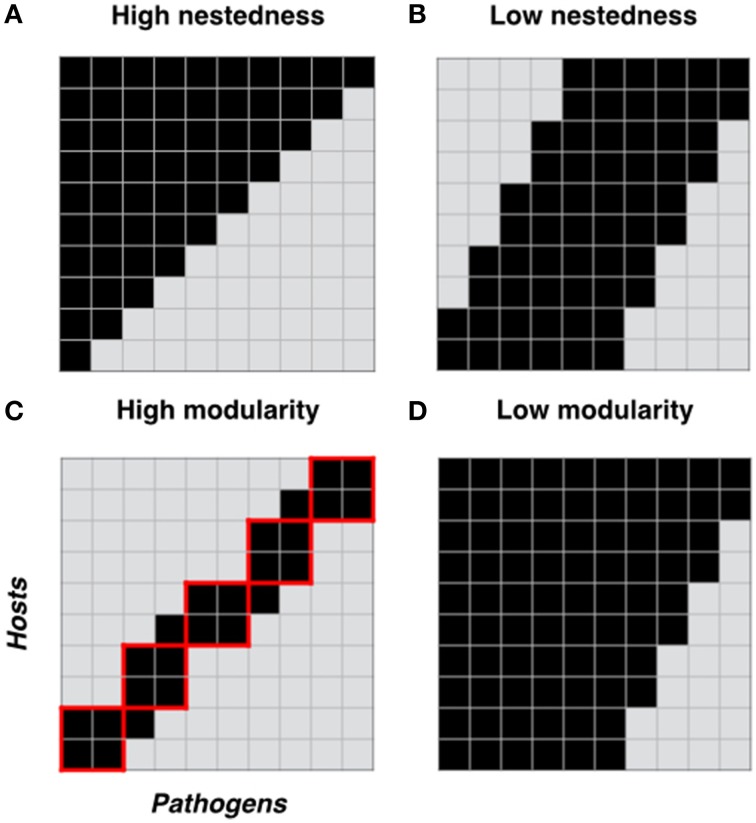
**Network structure properties of host-pathogen interactions**. Two important properties of ecological networks are nestedness and modularity. Here we show four cartoons representing different host-pathogen genetic interaction matrices, each with different levels of nestedness **(A, B)** and modularity **(C, D)**. For each matrix we show hosts in columns and pathogens in rows. Black squares in each matrix represent resistance between a plant and a pathogen genotype and gray squares represent host susceptibility. In **(C)**, red solid lines define host-pathogen interaction modules.

A network can be defined as a set of items, called nodes, connected by links if they interact. In host pathogen interactions, networks have two sets of nodes (each set representing individual hosts or pathogens) and are therefore termed “bipartite networks.” Links between nodes represent resistance or infection phenotypes (depending on the focus of the analysis). In such a network, specificity can be estimated for individuals or the network as a whole simply based on the number of links an individual has with its partners. In addition, patterns of specialization are often characterized by estimating two key network properties commonly known as nestedness and modularity (Figure 1). In host-pathogen networks, nestedness relates to the differentiation of resistance or infectivity specificities along a contained gradient, within which specialists (individuals with few links) interact with subsets of the partners interacting with generalists (individuals with many links) (Flores et al., 2012). For example, in a maximally nested infection network, the most specialized pathogen can infect only the hosts most susceptible to infection. The next most specialized pathogen could infect the host most susceptible to infection as well as one additional host, and so on (Figure 1A). Nestedness is a commonly encountered property in mutualistic networks (Jordano et al., 2003), and significant nested structures have been also found in studies of host-parasite (Vázquez et al., 2005; Vacher et al., 2008) and bacteria-phage (Flores et al., 2012; Poisot et al., 2013) networks. Modularity indicates the extent to which resistance or infectivity interactions can be partitioned into groups (referred to as modules: Figure 1C) with many interactions within groups but few among them (Blüthgen et al., 2008). In a maximally modular network there would be no cross-infections between pathogens in one module and hosts in another. Modularity differs from nestedness in that specificities cannot be simply ranked by increasing range. Rather, interactions take place among distinct clusters of host and pathogen individuals, within which distinct patterns of specificity (including nestedness) may be evident (Flores et al., 2012). Like nestedness, modularity is commonly detected in species interaction networks (Olesen et al., 2007; Fortuna et al., 2010).

**Key Network Properties:**

- **Nodes**: bipartite networks have two sets of nodes with each set representing individual hosts or pathogens.
- **Links**: resistance or infection phenotypes “linking” the nodes
- **Nestedness**: is a network structure describing a scenario where specialist individuals or genotypes (e.g., hosts with a very narrow resistance spectrum) interact with a subset of partners with which generalist individuals or genotypes (e.g., hosts with a very broad resistance spectrum) also interact (Bascompte et al., 2006).
- **Modularity**: A module (sometimes called a compartment) in a network is formed by a group of genotypes or individuals, which are more connected to one another than to individuals in other groups (Olesen et al., 2007). A modular network consists of a series of interconnected modules. In a host-pathogen network, modularity indicates the extent to which resistance or infectivity interactions can be partitioned into groups (referred to as modules: Figure 1C) with many links within groups but few among them.
- **Connectance**: is the proportion of links or realized interactions (p) over all possible interactions (P × H) (*C* = p/(P × H)).

Patterns of specialization can in turn be employed to make inferences regarding evolutionary dynamics, in that specific structures in host-pathogen networks may be predicted given different evolutionary scenarios. For example, modular patterns of specialization might be expected to emerge in networks where there is spatial or temporal segregation of host and pathogen genotypes (e.g., local adaptation; ecotypic divergence; cryptic speciation). Simply put, spatially or temporally co-occurring individuals within a network may be predicted to be more likely to fall within the same module, and the greater the evolutionary, spatial or temporal separation, the stronger the forces generating modularity should be. Nested patterns of specialization may likewise emerge under specific evolutionary conditions. For example, within a network constructed from a temporal sequence of interacting hosts and pathogen populations, patterns of nestedness emerging over time may be consistent with the classically envisaged stepwise coevolutionary arms race (Beckett and Williams, 2013) occurring via selective sweeps of novel resistance and infectivity alleles. Under this simple model in which pathogens evolve over time to expand their range of infectivity, and hosts counter-evolve to become more resistant, the host range of past pathogens should be sequentially nested within the host range of future pathogens. In other cases, spatial variation in the distribution of resistance and infectivity genotypes and fitness trade-offs (Thrall and Burdon, 2003) may maintain variation in specificity within or among populations, which may in turn result in promote the nesting of resistance and infectivity specificities.

The underlying genetic architecture of the interaction has also been predicted to be important in generating patterns of specificity and hence network structure (Flores et al., 2012; Moury et al., 2014). Several different models that describe the genetic nature of host-pathogen interactions have been proposed, the most important of which are the gene-for-gene (GFG) and matching allele (MA) models (see Thrall et al. this issue for review). The GFG model is commonly described in plant-pathogen associations and is generally well supported by phenotypic and genetic data as well as by a detailed mechanistic understanding of genes governing plant immune responses and pathogen infection (Dodds and Rathjen, 2010). In GFG systems, resistance is dependent upon plants producing a specific resistance gene product that recognizes specific pathogen elicitors (known as Avr genes). Pathogens that do not produce an elicitor recognized by a given R gene are able to infect. In contrast, the MA model describes an interaction where infection is dependent upon the pathogen carrying a gene or allele that is a specific match to a corresponding host genotype. Hosts without the recognized allele are resistant (Thrall et al. this issue). All else being equal, the topology of resistance and infectivity matrices (i.e., patterns of nestedness and modularity) is likely to more or less reflect underlying genetic interaction. Under a GFG model with additive variation in the number of genes or alleles conferring additional resistance or infectivity specificities, cross-infectivity, where a parasite with a given genotype is able to infect hosts with different genotypes, is common (Thrall and Burdon, 2002). Pathogen mutations conferring new infectivity do not necessarily result in recognition by an alternative R gene. The same is true for host resistance. Hence, there is strong potential for the maintenance of variation in partner breadth within GFG interactions, and the genetic interaction matrix is seemingly naturally nested (Thrall et al. this issue). In contrast, under the MA model, pathogen mutations conferring new infectivity simultaneously result in the emergence of resistance in existing host genotypes. Hence, cross-infectivity is rare, and the interaction matrix is inherently modular (Moury et al., 2014).

The main aim of this paper is to apply network based approaches to investigate patterns of specialization within spatial and temporal plant-host fungal-pathogen interaction (i.e., resistance/infection) networks. *Linum marginale* is a perennial herb endemic to southern, temperate areas in Australia. *Melampsora lini* is an autoecious, macrocyclic foliar rust pathogen that is restricted in Australia to *L. marginale*. Utilizing existing data from the *M. lini*-*L. marginale* interaction, network structure in resistance and infectivity networks can be investigated in a relatively extensive, balanced and rigorous way. Disease outcomes in this system are governed by a GFG interaction and extensive polymorphisms for host resistance and pathogen infectivity form the basis for selective changes in host and pathogen populations (Burdon, 1994; Barrett et al., 2009b; Thrall et al., 2012). Furthermore, sampling can readily be done across a range of defined temporal and spatial scales, and the system is well characterized from various ecological and evolutionary perspectives. The pathogen has substantial fitness effects on the host, with 60–80% reductions in population size documented during severe epidemics (Jarosz and Burdon, 1992). Plants recruit via predominantly self-fertilized seed (Burdon et al., 1999) and the pathogen is almost exclusively clonal within the region from which all pathogens used in this study were collected (Barrett et al., 2008). Previous work has established roles for local adaptation (Thrall et al., 2002), gene-flow (Barrett et al., 2008), trade-offs (Thrall and Burdon, 2003), diversifying selection (van der Merwe et al., 2009), and spatio-temporal dynamics (Thrall et al., 2012) in driving evolutionary change in interacting host and pathogen populations. The results of these studies provide clear evidence: (i) of a genetic basis for race-specific resistance and infectivity; (ii) that there is variation among individuals for these traits within and among populations; and (iii) that pathogen attack impairs host fitness.

To examine how patterns of specialization in infectivity and resistance develop in the *L. marginale*-*M. lini* interaction we analyzed data networks collected as part of previous investigations of spatial and temporal evolutionary dynamics. Specifically, we analyzed data from two spatial (Thrall et al., 2002; Laine et al., 2014) and six temporal interaction networks (Thrall et al., 2012). We asked: Do patterns of specificity vary across different networks within the same interaction? Are there significant modules and nested structures within different spatial and temporal networks? Are these related to spatial and temporal segregation of interacting genotypes? How do patterns of specificity change across different networks and can drivers of change be identified?

## Materials and methods

### Datasets

To investigate and compare spatial and temporal variation in infectivity and resistance in the *L. marginale-M. lini* interaction we utilized datasets described in three previously published papers (Table 1: Thrall et al., 2002, 2012; Laine et al., 2014). Sampling methodologies and experimental techniques were very similar across all of these studies. In each case, host and pathogen materials were collected from spatially discrete populations in open, subalpine grasslands within Kosciuzko National Park, NSW (the number of individuals sampled varied depending on the original study). For each study, host and pathogen collections were timed to coincide with the peak epidemic period (January–Febuary, in the year of collection). At each site, host seed (maternal families) was collected from multiple unique *L. marginale* individuals (in this area *L. marginale* is tightly inbreeding; Burdon et al., 1999). At the same time, single pustules of *M. lini* were sampled haphazardly, each from a single pustule on a unique infected plant. After collection each pathogen isolate was purified though a process of single-pustule isolation to ensure genetic purity. Each sample was then put through multiple rounds of increase on a universally susceptible *L. usitatissimum* c.v. Hoshangabad plant, vacuum dried and stored at 5C until the beginning of the reciprocal inoculation experiments.

**Table 1:** **Summary of datasets used in this study for assessing spatial and temporal patterns of specialization in host-pathogen interaction networks**.

**Reference**	**Focus**	**Name (abbreviation)**	**Scale**	**Size of Matrix (s)**	**Reciprocity**
Thrall et al., 2002	Local adaptation network	Local Adaptation (LA)	Six populations, single timepoint	60 pathogen × 120 host matrix	Fully reciprocal among all localities
Laine et al., 2014	Local and ecotypic adaptation network	Bogs-Hills (BH)	Two ecotypes, four populations per ecotype, single timepoint	80 pathogen × 73 host	Fully reciprocal among all localities and ecotypes.
Thrall et al., 2012	Six networks examining temporal change and adaptation	Kiandra (K); N1; N2; B1; B2; B3	Six populations, four timepoints within each population	40 pathogen × 60 host for each of 6 networks	Fully reciprocal among years within populations. No among population testing

To perform reciprocal inoculation experiments, host plants were propagated (one seed per maternal family) until they reached a suitable size to provide cuttings. Shoots were then cut from host plants and placed in water-filled tubs. Each tub included a cutting of the fully susceptible *L. usitatissimum* c.v. Hoshangabad to confirm pathogen viability. Tubs containing shoots of 14 host lines plus the control were inoculated with approximately 10 mg of spores from a *M. lini* isolate. The following day, tubs were transferred to a naturally lit greenhouse and infection was scored 12–14 days later. Recorded infection types were: 1 = fully susceptible [large sporulating pustules (uredia) on all leaves]; 2 = partial resistance (large sporulating pustules on younger leaves only, with no pustules on the oldest leaves); 3 = partial resistance (large pustules only on one or two of the youngest leaves); 4 = partial resistance (no sporulation, but with necrotic flecks on older leaves); 5 = full resistance (fully incompatible reaction with no macroscopic evidence of damage or sporulation). Previous genetic studies suggest that both full and partial resistance reactions are race-specific and likely under the control of single, dominant genes for resistance in the host (Burdon, 1994).

The dataset of Thrall et al. (2002) includes hosts and pathogens collected from six localities and were designed to test for pathogen local adaptation. The six populations can be subdivided into three spatial groups; northern (GI, G3) and southern (SHI, SH2) Kiandra Plain; and Wild Horse Plain (WHP1, WHP2). The northern and southern population groups are approximately 10 km apart, and are 6.1 km and 4.6 km from the group located on Wild Horse Plain, respectively. Within each group, interpopulation distances average 460 m. Resistance and infectivity structure of host and pathogen populations was determined in a fully reciprocal fashion by exposing 20 plant lines to 10 pathogen isolates for each of the six populations, giving a total of 7200 individual inoculation tests. This dataset is subsequently referred to as the LA network.

The dataset of Laine et al. (2014) includes hosts and pathogens collected from eight localities and were designed to test for local adaptation and ecotypic divergence. Four of these populations (CBL Hill, CEM, G3, and SH2) occurred on slopes and are referred to as hill populations. Four populations (CBL Bog, G2, P1, and PS) were on flatter bog habitats and are called bog populations. The plants from these two habitat types can be distinguished by their morphology and are also genetically differentiated (Thrall et al., 2001), thus they are considered to represent different plant ecotypes. The pathogens from the two habitat types cannot be distinguished morphologically, but are genetically differentiated and are likewise considered different ecotypes. The study populations are situated in three areas of the Kosciuszko region that are separated by some tens of kilometers. Fully reciprocal inoculation testing involved 10 host lines and 10 pathogen isolates chosen from each of the populations, giving a total of 6400 individual inoculation tests (in the final analysis, 7 host lines were removed owing to low germination and hence high levels of missing data). This dataset is subsequently referred to as the BH network.

The dataset of Thrall et al. (2012) used host and pathogen materials collected from six localities in the general area of the Kiandra Plain in the Kosciusko region. The experimental design in that study was focused on studying temporal change *within* populations. Unlike the two spatial datasets (Thrall et al., 2002; Laine et al., 2014), no reciprocal inoculation testing was performed *among* populations, thus each population is treated as a separate network here. Fully reciprocal inoculation testing (host *by* pathogen *by* year) within each population was conducted over three time points for the host (2004, 2006, and 2008) and four time points for the pathogen (2002, 2004, 2006, and 2008), to give a total of 2400 pairwise inoculations per population and 14,400 overall.

### Data analyses

#### Data transformations

In this manuscript we analyzed data largely from the perspective of host resistance (as opposed to pathogen infectivity). For graphical analyses infection type 1 plants were classified as susceptible; infection type 2 and 3 plants were classified as partially resistant, and infection type 4 and 5 plants were classified as fully resistant. For all other analyses, we utilized only binary information: infection type 1 plants were classified as susceptible, and all other infection reactions were classified as resistant. Although the use of binary data resulted in the loss of information from some networks, the presence of only single (partial) resistance types in some networks meant that both qualitative and quantitative methods would have been required to generate nestedness values, run null models analyses and perform comparative analyses.

#### Specificity and general network structure

For each network, we calculated resistance structure (% full resistance; % partial resistance; % full susceptible; % resistance of any kind). Resistance breadth is simply the proportion of pathogens to which a host displays resistance. The resistance specificity was summarized for each network by calculating a resistance specificity (Rs) index:

(1) $$\begin{array}{l} Rs=\frac{P\;-\;r}{P\;-\;1} \end{array}$$

where *P* is the number of pathogens to which the host has been exposed, and *r* is the sum of the resistance reactions (both full and partial). Hosts able to resist all pathogens in the network (generalists) have a value of 0, while hosts able to resist only a single pathogen (specialists) have a value of 1. To generate a network level summary statistic this was calculated for each host in the network and then averaged for all hosts.

#### Nestedness

We assessed the nestedness of each individual network visually and by using the Nestedness based on Overlap and Decreasing Fill index (NODF) (Almeida-Neto et al., 2008). In the context of resistance, NODF measures the extent to which hosts resist a subset of the pathogens resisted by another more widely resistant host; NODF values of 100 indicates the matrix is perfectly nested and a value of 0 means that it is not nested at all. NODF is sensitive to the connectance (i.e., total number of links or resistance reactions) of the input matrix (Almeida-Neto et al., 2008). Because connectance is variable among networks (Table 2), this makes direct comparison among networks problematic. Observed values of nestedness therefore need to be interpreted in the context of expected values of nestedness derived from a null distribution of matrices generated by more or less randomly rearranging the parent matrix (Ulrich et al., 2009). NODF values were calculated, and null model comparisons were performed using the R package bipartite (Dormann et al., 2009). For comparison among all networks, matrices were sorted to maximize nestedness prior to NODF being calculated. We generated 10,000 null models for each network using the mgen method for binary matrices (Dormann et al., 2009; Vázquez et al., 2009). The mgen model returns a random web based on the number of links in the parent network and is otherwise unconstrained (i.e., all pairwise interactions have the same probability of occurrence). Z scores were used to compare among NODF values calculated for the observed network vs. the distributions of values calculated for the null models. In addition, for the temporal networks, we calculated NODF values for each matrix with the ordering of rows and columns reflecting temporal ordering (as opposed to sorting to maximize nestedness in the previous analyses). This facilitates a test of the hypothesis that nested resistance structures arise as a consequence of temporal coevolutionary dynamics (Ulrich et al., 2009).

**Table 2:** **General properties of the *Linum marginale-Melampsora lini* spatial and temporal networks**.

**Properties**	**Spatial networks**		**Temporal networks**					
	**LA**	**BH**	**K**	**N1**	**N2**	**B1**	**B2**	**B3**
Number hosts	120	73	60	60	60	60	60	66
Number pathogens	60	80	40	40	40	40	40	40
Network size	7200	5840	2400	2400	2400	2400	2400	2640
% full resistance	10.2	6.3	11.8	0	0	0	0	3.5
% partial resistance	14.8	25.8	17.2	42	45.5	78	42.4	37
Connectance	0.25	0.32	0.29	0.58	0.46	0.78	0.42	0.4
% full susceptible	75	67.8	71	58	54.5	22	57.6	59.5
Resistance specificity	0.763	0.69	0.73	0.43	0.56	0.22	0.59	0.61
Proportional susceptibility	0.90	0.94	0.88	1	1	1	1	0.96

*See Table 1 for additional information on study design. Network properties are defined in text in the M&M*.

#### Modularity

A modular network consists of interconnected modules. Each module is formed by a group of genotypes or individuals, which are more connected (i.e., have similar resistance phenotypes) to one another than to individuals in other groups (Olesen et al., 2007). We used the *simulated annealing* algorithm (SA) (Guimera and Amaral, 2005) to estimate the level of modularity (M). Basically, M is a measure of the extent to which individuals have more links within their modules than expected if linkage is random. The SA algorithm identifies the modules present in the networks, whose nodes have most of their links (or interactions) inside their own module (Guimera and Amaral, 2005). Almost all nodes are unambiguously assigned to a module, except extreme connector nodes, i.e., individuals equally connected to several modules. For each network, SA calculates an index of modularity *M*:

(2) $$\begin{array}{l} M=\;\overset{N_{M}}{\underset{s=1}{\sum }}\left({\frac{I_{S}}{I}\;-\;\left(\frac{K_{S}}{2I}\right)}^{2}\right) \end{array}$$

where *N*_*m*_ is the number of modules in the network, *I*_*s*_ is the number of links between hosts and pathogens within module *s, I* is the number of links in the network, and *k*_*s*_ is the sum of degrees of all individuals in *s*. For each empirical network, we did an SA analysis of 100 random networks with the same nodes degree distribution as the empirical one, and examined whether the empirical network was significantly more modular than the random ones (Guimera et al., 2004). Thus, we calculated a z-score value and its significance (α = 0.05) to estimate whether a network was significantly modular or not. In addition, to test if modularity was influenced by sampling location or year we used log-linear models to analyse count data according to assigned module and sampling location/time using the glm() function in R.

## Results

We analyzed resistance data from a total of 8 host-pathogen networks comprising: (1) two host-pathogen networks (LA, BH) based on spatial variation (i.e., fully reciprocal testing among populations) and (2) six host-pathogen networks based on temporal variation (i.e., fully reciprocal testing among years but only within local populations).

### Resistance specificity and general network properties

**Figure 2:**
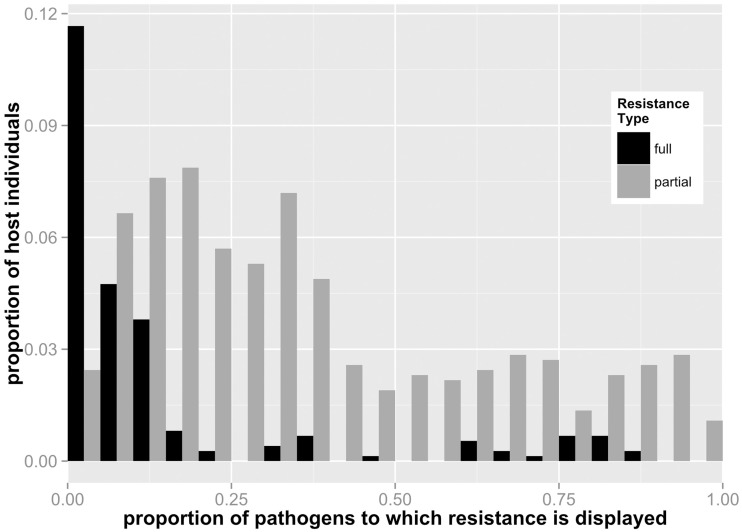
**Frequency distribution of full and partial resistance specificities for individual host lines for all ***Linum-Melampsora*** networks (i.e. merged datasets)**. For example, a value of 0.5 for full resistance means that an individual host displays full resistance to 50% of pathogens to which it was exposed. This figure demonstrates that generalist hosts are more likely to express partial resistance.

**Figure 3:**
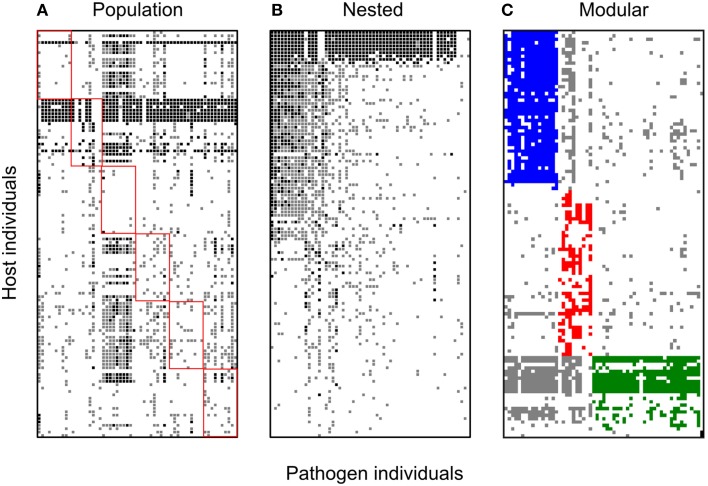
**Bipartite spatial networks for the ***Linum-Melampsora*** local adaptation dataset**. Black squares indicate full resistance, gray squares indicate partial resistance, and white squares indicate full susceptibility. Hosts are represented on the Y-axis and pathogens on the X. All panels are formed from the same base dataset but sorted in different ways so as to facilitate visually testing different network hypotheses: **(A)** sorted by population of origin. Host and pathogen interactions corresponding to the same population are indicated by red squares; **(B)** sorted to maximize nestedness; and **(C)** sorted to maximize modularity. The network is significantly nested (NODF; *P* < 0.001). For the modularity sorting, discrete modules are shown in different colors (blue, red, green, black). Four modules could be identified in this network with the *P*-value for observed modularity less than 0.0001.

**Figure 4:**
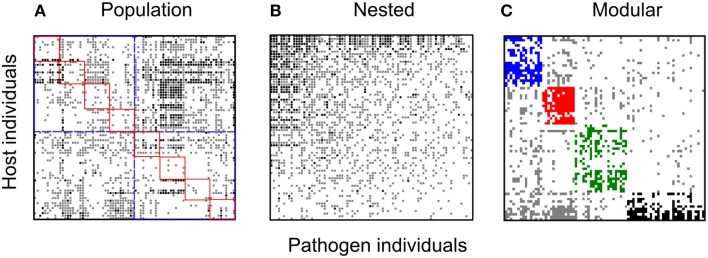
**Bipartite spatial networks for the for the ***Linum-Melampsora*** BH network**. Black squares indicate full resistance, gray squares indicate partial resistance, and white squares indicate full susceptibility. All panels are formed from the same base dataset but sorted in different ways so as to test different network hypotheses: **(A)** sorted by population and ecotype of origin. Host and pathogen interactions corresponding to the same population are indicated by red squares while the ecotypes are shown by the blue squares; **(B)** sorted to maximize nestedness; and **(C)** sorted to maximize modularity. For nestedness, the network is significantly nested (NODF; *P* < 0.001). For the modularity sorting, discrete modules are shown in different colors (blue, red, green, black). Four modules could be identified in this network with the *P*-value for observed modularity less than 0.0001.

General properties of the host-pathogen networks are shown in Table 2. All networks were highly variable for resistance patterns among individual hosts, both for full and partial resistance types. Considering all resistance types, resistance networks were moderately connected but skewed toward full susceptibility (Table 2). For all eight networks, we observed a continuum of individual strategies with regards to the breadth of resistance (Figures 2–**4**), ranging from fully susceptible (i.e., no resistance) though to full or partial resistance to the large majority of pathogens in the network. In terms of pathogen infection (i.e., both partially resistant and fully susceptible hosts), individual hosts on average were susceptible to infection from a very high proportion of pathogens in the network (88–100%: Table 2).

Total and relative levels of full and partial resistance varied widely among networks (Table 2). Four of the temporal networks displayed no full resistance reactions at all (i.e., all resistance was partial: N1, N2, B1, B2), while a fifth (B3) displayed a relatively low ratio of full vs. partial resistant plants compared to the remaining temporal (K) and spatial networks (LA, BH). Resistance specificity also varied markedly between networks (Table 2). We explored the relationship between specificity and the incidence of full and partial resistance at both the individual and whole network levels. For individual hosts, we counted the number of pathogens targeted by full vs. partial resistance. At this individual level, full resistance was triggered by a relatively small number of pathogens in the network), while the range of pathogens targeted by individuals with partial resistance spanned a wider and more even spectrum (Figure 2). For example, on average, the full resistance carried by any individual host plant was effective against 14.2% of pathogens, while partial resistance was effective against 37.3%.

### Nestedness

**Figure 5:**
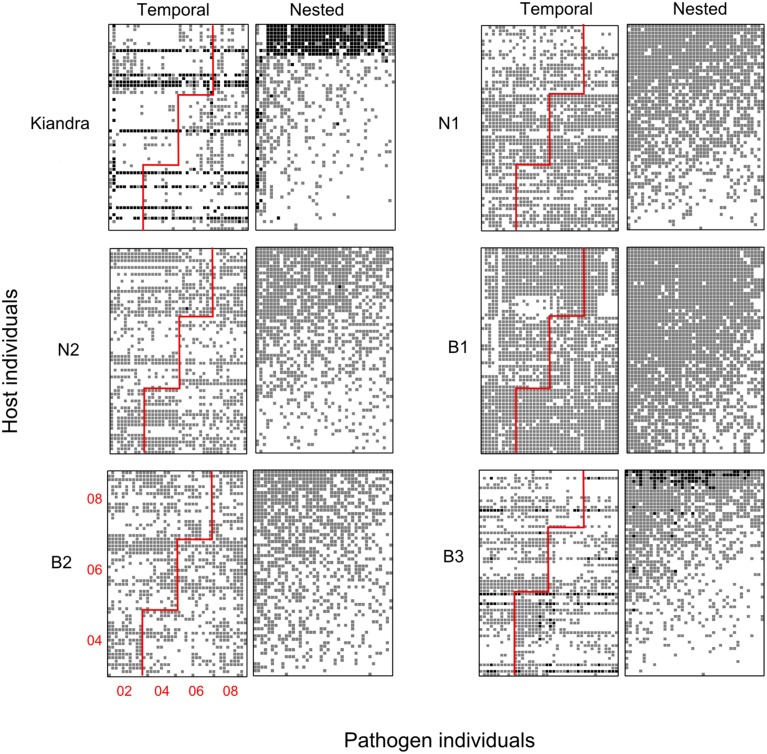
**Bipartite networks for the ***Linum-Melampsora*** temporal datasets**. Black squares indicate full resistance, gray squares indicate partial resistance, and white squares indicate full susceptibility. Each pair of panels represents a separate population/temporal network (labeled Kiandra, N1, etc.). The two columns for each population pair are formed from the same base dataset but sorted in different ways so as to facilitate visually testing different evolutionary hypotheses. The left panels have been sorted according to sampling period (i.e., temporally), while the panels to the right have been sorted to maximize nestedness. For the temporally sorted networks, the red line demarks contemporary host and pathogens, such that an interactions above and to the left of the red line indicate hosts interacting with past pathogens (year of sampling is shown in the bottom left panel). The expectation under an arms race hypothesis is that there should be a nested pattern emerging over time, such that more resistance reactions occur above and to the left of the red line. All networks were significantly nested when sorted to maximize nestedness (Table 3: NODF; *P* < 0.05), but none were significantly nested under the temporal arrangement.

When matrices were sorted to maximize nestedness, values of NODF were significantly higher than expected by chance for all networks (Table 3) and all networks visually appeared to be nested (Figures 3–5). This indicates that for all 8 networks, resistance specificities displayed by hosts with a wide range of resistance (generalists) tended to encompass resistance specificities displayed by hosts with a narrow range of resistance (specialists). In addition, we note that in networks with full resistance types, generalist hosts tended to carry relatively high levels of full resistance (Figures 3, 4; although this pattern is less evident in the BH network).

**Table 3:** **Nestedness metrics (NODF index) as compared to null model distributions for ***Linum marginale-Melampsora lini*** networks sorted to maximize their nested structure**.

**Network**	**Type**	**NODF**	***z*-score**	***p*-value**
LA	Spatial	49.68	6.36	< 0.0001
BH	Spatial	44.1	3.58	0.00034
K	Temporal	51.02	5.86	< 0.0001
N1	Temporal	72.44	6.27	< 0.0001
N2	Temporal	58.30	2.79	0.00527
B1	Temporal	84.20	7.41	< 0.0001
B2	Temporal	54.10	2.09	0.03662
B3	Temporal	63.00	5.93	< 0.0001

*In all cases NODF values were higher than expected by chance. P-values lower than 0.05 were interpreted as indicating that observed nestedness was greater than expected by chance*.

With regards to evolutionary drivers of nestedness, when temporal matrices were ordered according to year of sampling (rather than re-ordered to maximize nestedness), the K and B3 networks displayed significantly higher values of nestedness (*z* = 4.27 and 2.71, respectively) than those calculated for corresponding unsorted null model matrices (Table 4). However, visually it would seem that specialist and generalist hosts were not distributed through time in any predictable or clustered way (Figure 5), and values of NODF were much lower than those calculated for network and null model matrices sorted to maximize nestedness (Tables 3, 4). For the spatial networks, there was an obvious spatial component to patterns of nestedness and resistance breadth (Figure 3). In other words, and as noted in previous studies (Thrall and Burdon, 2003; Laine et al., 2014) specialist and generalist types were not randomly distributed among populations. For example, for the LA network, the 10 most generalist hosts were all sampled from local population G3 (known to be highly resistant from previous studies; Thrall et al., 2002), while 10 of the 20 most specialized hosts were sampled from the generally susceptible population SH1 (data not shown).

**Table 4:** **Nestedness metrics (NODF) as compared to null model distributions for temporal ***Linum marginale-Melampsora lini*** networks sorted by year of sampling**.

**Network**	**NODF**	***z*-score unsorted**	***z*-score 2 sorted**
K	26.11	4.28	−14.5
N1	40.73	1.82	not calculated
N2	24.9	−0.32	not calculated
B1	32.7	−0.10	not calculated
B2	30.8	1.03	not calculated
B3	31.8	2.71	−24.93

*For K and B3 networks, Z-scores are higher than expected by chance in unsorted matrices. However, they are significantly less nested then expected by chance when random matrices are sorted to maximize nestedness (z-score 2)*.

### Modularity

All eight networks (six from the temporal variation data set and two from local adaptation data sets) were analyzed separately. For the temporal variation networks only one (Kiandra) was significantly modular. In contrast, the resistance networks from both spatial datasets (LA and BH) showed significant levels of modularity (see Table 5) and the same number of modules (*n* = 4). Module size (number of hosts and pathogens comprising the module) also did not vary greatly (Figures 3, 4), with the exception of one module within the LA network which had only three nodes (Figure 3). In terms of number of links within modules, we found high levels of variation among modules within the LA network where some modules had high within-module connectivity and other modules had more links with other modules (thus serving as connectors). In the BH dataset, all modules had a similar number of links within modules as well as connections to other modules (data not shown).

**Table 5:** **Modularity analysis statistics based on ***n*** = 100 randomizations for all networks studied (α = 0.05)**.

**Network**	**Modularity**	***z*-score**	***p*-value**
LA	*M* = 0.275	*Z* = 38.62	< 0.0001
BH	*M* = 0.297	*Z* = 25.29	< 0.0001
B1	*M* = 0.065	*Z* = −17.9	< 0.0001 (significantly less modular)
B2	*M* = 0.15	*Z* = −4.16	< 0.05 (significantly less modular)
B3	*M* = 0.15	*Z* = −0.88	> 0.05
Kiandra	*M* = 0.23	*Z* = 7.61	< 0.05
N1	*M* = 0.089	*Z* = −13.4	< 0.05 (significantly less modular)
N2	*M* = 0.143	*Z* = −3.1	< 0.05 (significantly less modular)

*Negative z-score values indicate that the mean modularity of randomized matrices was higher than the observed modularity, and some of these were significantly less modular than randomized networks*.

With regards to evolutionary drivers of modularity, for the spatial networks we hypothesized that modules would comprise hosts and pathogens sourced from either the same geographical location (or ecological habitat in the case of the BH network). As predicted for the BH dataset, ecological habitat (GLM; *p* < 0.0001) was an important predictor of host modularity (Table 6; Figure 2). We found three modules mainly composed of host individuals occurring in populations of the hills ecotype (Modules 1, 2, and 4) and one other module largely comprising individuals collected from populations within bog habitats (Module 3). More specifically, modules 1 and 2 largely (although not exclusively) comprised hosts with a hills origin (75 and 87%, respectively) with specific resistance to a subset of bog pathogens (73 and 100%, respectively); module 3 in contrast comprised individuals collected from bog habitats with more specific resistance to a group comprising largely hills pathogens (86%); and module 4 comprised a highly resistant set of hills hosts interacting with a mixture of otherwise highly infective pathogens (55% hills origin). Furthermore, the distribution of hosts assigned to different modules was also significantly influenced by population within ecotype (GLM; *p* < 0.01). For example, half of the individuals assigned to module 2 were collected from the CBL Hill population (Table 6). In the LA network, while the frequency of individuals assigned to different modules was variable among sampling sites, modules were mostly composed of a mix of individuals sourced from different cohorts (data not shown), and we found no significant support for the hypothesis that modules comprise individuals from specific populations or sets of populations (GLM; *p* > 0.05). For the temporal dataset, only one (Kiandra, *M* = 0.23) out of six networks had a significantly modular host-pathogen resistance network. We found four modules in the Kiandra network and each module was mostly composed of a mix of hosts and pathogens sampled from different time points (results not shown).

**Table 6:** **Number of host individuals from different ecotypes and populations assigned to resistance modules in Bogs-Hills network**.

**Ecotype**	**Population**	**Module1**	**Module2**	**Module3**	**Module4**
Hill	Cemetery	5	2	0	3
	G3	4	0	0	5
	CBL Hill	0	8	1	1
	SH2	6	3	0	0
Bog	G2	2	0	7	1
	P1	3	0	5	1
	CBL Bog	0	2	6	0
	PS	0	0	8	0

*See Laine et al. (2014) for a map showing relative locations of these populations*.

## Discussion

### Summary of major results

Genetic variation for resistance and infectivity are ubiquitous in wild populations of plants and their associated pathogens (Laine et al., 2011; Tack et al., 2012). However, many questions remain regarding the ecological and evolutionary processes that generate and maintain such variation. In this study we used a network analytical approach to examine the architecture of host-pathogen interactions through space and time using several extensive datasets from a longstanding wild plant-pathogen system. We found consistent, and in some cases, strong patterns in the data. First, we found that a spectrum of specialist and generalist resistance (or infectivity) types are consistently maintained within these networks and that partially resistant host are more likely to have a broad resistance spectrum. Second, we found that all networks were significantly nested. Third, we found that resistance networks were significantly modular in both spatial networks, but in only one of the six temporal networks. These results demonstrate that network approaches have potential to complement more commonly used approaches to analysing population and temporal-based sampling by placing a more explicit focus on individual variation in patterns of specificity, and revealing structure (i.e., nestedness and modularity) in the data that are independent of common *a priori* hypotheses (e.g., local adaption). Below, we discuss how the topology of *L. marginale-M. lini* interaction networks has the potential to provide novel insight into the evolution of genetic interactions that underpin disease outcomes within this host-pathogen association.

### Spatial dynamics and network structure

Spatial evolutionary processes (e.g., local adaptation of pathogens to their hosts) have been demonstrated to be important drivers of genetic structure and specialization in several plant host–pathogen interactions (see Barrett et al., 2009a for review). Here we investigated the idea that patterns of modularity within networks have potential to reveal the strength, scale, and direction of spatial (co)evolutionary processes. In particular, given small, geographically, and genetically differentiated *L. marginale* and *M. lini* populations (e.g., Barrett et al., 2008; Nemri et al., 2012; Thrall et al., 2012), and previously reported patterns of local adaptation in this system (Thrall et al., 2002; Laine et al., 2014) we hypothesized that modularity should be apparent in the two spatial networks (i.e., LA and BH) and that there should be clear links between population structure and patterns of modularity.

For the BH network, ecotype emerged as a strong predictor of modularity. In particular, for the four modules detected, one module almost exclusively comprised bog hosts resistant to hill pathogens, while the three remaining modules comprised mostly hill hosts resistant to bog pathogens. At the population level (i.e., within each ecotype), the locality from which individuals were collected was also a significant predictor of modularity, although divisions were not as clear as for ecotype. These results are consistent with Laine et al. (2014) who demonstrated using the same dataset that hosts were more likely to be resistant to pathogens from a different host-ecotype, and conclude that habitat type is a strong driver of evolutionary divergence among both hosts and pathogens. The results also support those of Flores et al. (2012) found that modularity in a bacteria-phage network was driven in part by geographic structure, suggesting that geography may be an important determinant of modularity generally. However, it should be noted that resistance across habitat types was also observed frequently (e.g., generalist hosts with wide ranging resistance), and modules typically contained hosts and pathogens from both habitats. In contrast to the BH network, there was no obvious geographic signal in the LA network. Rather, modules were mostly composed of individuals sourced from a mix of different populations (geographical locations) despite the strong evidence for local adaptation (Thrall et al., 2002). Hence, while our results are consistent with the idea that spatial evolutionary dynamics may result in the emergence of modularity, with regards to our data it seems that spatial structure may only be sufficient under some conditions. For example, the ecotypic differentiation among hill and bog environments may well result in stronger barriers to gene flow and stronger local spatial selection gradients than in the LA network which included populations within the hill ecotype only. Local geographic separation (as is the case for the LA network) may not be enough to drive the evolution (or maintenance) of significant modularity at the local metapopulation scale where ecological barriers to gene flow are weak or non-existent and other selective forces are at play (e.g., Thrall and Burdon, 2003).

### Temporal dynamics and network structure

Temporal coevolutionary processes have been widely hypothesized and in some cases demonstrated to be important drivers of genetic structure and specialization in host–pathogen interactions (Decaestecker et al., 2007). We investigated whether networks constructed from temporal sequences of interacting host and pathogen populations displayed patterns of change in nestedness or modularity over time (Beckett and Williams, 2013). We found little evidence for consistent temporal changes in either metric, despite findings supporting reciprocal coevolution in a previous study (Thrall et al., 2012). Significant modularity was evident in only one of the six temporal networks, and different modules in the Kiandra network were composed of various individuals collected from a mixture of different time-points. In addition, while we found significant patterns of nestedness in all temporal networks, there was little evidence to support the hypothesis that temporal patterns of genetic change were responsible for generating them. In particular, no patterns of increasing nestedness or even generality over time were evident in the data (e.g., with respect to host resistance to pathogens sampled from previous time points). This suggests that temporal evolutionary processes, at least over the time-frames, within which we sampled, are insufficient for generating clear patterns of either nestedness or modularity within the host-pathogen networks sampled in this study. However, it should be noted that *L. marginale* is a short-lived perennial host (approximately 5 years) and while pathogen can have substantial fitness effects on the host, disease epidemics do not predictably occur in all years or populations. As for the spatial evolutionary scenarios discussed above, ecological and evolutionary patterns are strongly dependent on the scale of inquiry as well as host and pathogen life-history, and in this case, it is possible that patterns may only become evident for comparisons that encompass longer time periods. Furthermore, these results should also be interpreted in light of the high levels of partial resistance that we found in 5 of the 6 temporal networks, in that selection pressures may be more diffuse in these situations given the lower levels of resistance specificity maintained (Antonovics et al., 2011).

### Mechanisms determining nestedness

Considering the variation in sampling scale, the repeated finding of nested patterns of specialization across networks suggests this is a universal pattern for *L. marginale-M. lini* interaction networks. Because utilization of network approaches in studies of patterns of infectivity and resistance within host-pathogen interactions is still in its infancy, it is difficult to speculate on the comparative significance of these results. However, in one of the few other large scale studies of network structure in host-pathogen interactions, Flores et al. (2011) report consistent, significant patterns of nestedness across 38 bacteria-phage networks, suggesting that nestedness may be a common property of antagonistic interaction networks. However, the processes generating nested structures in our system (or the phage networks) are not obvious and may be attributable to several non-exclusive mechanisms.

One parsimonious explanation is that the underlying genetic architecture of the interaction may constrain networks to a nested shape (Flores et al., 2012; Moury et al., 2014). In GFG systems such as the *L. marginale-M. lini* interaction, qualitative patterns of resistance such as those reported in this study are dependent upon plants producing a specific resistance gene product that recognizes specific pathogen elicitors. Assuming that loss of the elicitor does not enable activation of another R gene or otherwise compromise infectivity, pathogens that do not produce a recognized elicitor are able to infect. Assuming additive variation in the number of genes or alleles conferring additional resistance or infectivity specificities, cross-infectivity, where a pathogen with a given genotype is able to infect hosts with different genotypes, should be common (Thrall and Burdon, 2002). Hence GFG systems generate strong potential for variation in partner specificity, and may naturally generate nested interaction networks (Moury et al., 2014). A related and non-exclusive mechanism involves fitness trade-offs. In particular, pleiotropic costs of maintaining multiple R genes, or alleles that confer multiple specificities, may also be important in explaining the consistent maintenance of variation in resistance breadth (Barrett and Heil, 2012). Costs of resistance (e.g., Karasov et al., 2014) and infectivity (e.g., Barrett et al., 2011) have been demonstrated in several plant-pathogen interactions, including for *M. lini*, where trade-offs between pathogen host-range and spore production have been demonstrated (Thrall and Burdon, 2003). Certainly trade-offs provide a general explanation for the maintenance of specialist resistance types in the face of pathogen induced morbidity and mortality. Finally, it has also been proposed that nestedness may be a reflection of ongoing and dynamic evolutionary processes that typify antagonistic species interactions, such that hosts generally adapt to new infectivity genes or alleles without losing their specificity for older forms of infectivity (Flores et al., 2011). However, while nested patterns may potentially emerge over long-term time scales, results from our temporal networks (as discussed above) are not consistent with such dynamics generating these patterns on shorter time scales.

### Mechanisms determining modularity

While our results for the BH network are consistent with the idea that spatial evolutionary dynamics can drive the emergence of modularity (as discussed above), results from the LA and K (temporal) networks demonstrate that modularity can transcend spatial (and temporal) structure. One likely common determinant of modularity within host-pathogen interaction networks is genetic divergence among groups of individuals comprising one or both nodes in the network (Flores et al., 2011; Weitz et al., 2013). This also serves as a potentially general explanation for our findings of modularity across the LA, BH, and K networks. Certainly, in the bog-hill populations, ecotypic structure also reflects strong patterns of genetic divergence between hosts (Thrall et al., 2001) and pathogens (Laine et al., 2014; LG Barrett unpublished data). In addition, previous population genetic work demonstrates the maintenance of multiple clonal lineages of *M. lini* throughout the region where sampling for all of these studies was conducted (Barrett et al., 2008). Importantly, although there was some signature of population-level differentiation, different lineages were not confined to individual populations (Barrett et al., 2008). Therefore, it is possible that modularity in the LA and K networks may also reflect underlying genetic heterogeneities within and among the pathogen populations (and potentially hosts) from which the individuals comprising these networks were sampled. We suggest that for future studies, the generation of complementary population genetic data when examining resistance and infection networks could likely help reveal the proximate source of modular resistance structure.

### Partial resistance

One of the novel results emerging from this study was the finding that broad resistance specificities were more likely to be conferred via partial resistance. Importantly, the expression of partial resistance was still dependent on host-pathogen genotype interactions (i.e., partial resistance is race-specific), consistent with previous studies suggesting that partial resistance is under GFG control (Burdon, 1994). This result has interesting implications for our understanding of the factors that drive the evolution of specificity and resistance strategies and can perhaps most obviously be explained via a trade-off between host range and the strength of resistance. Negative genetic correlations among resistance strategies have been demonstrated in some previous studies (e.g., between induced and constitutive resistance), suggesting that pleiotropy or the otherwise costly expression of linked resistance traits may be common (Agrawal et al., 2010; Rasmann et al., 2015). However, a mechanistic understanding of how such trade-offs arise is generally lacking. While we do not have any data that speaks directly to the mechanisms that may underlie a trade-off between specificity and the strength of the resistance response, a recent study using the interaction between *Melampsora lini* and *Linum usitatissimum* shows that the recognition of pathogen elicitors and the strength of the subsequent response are intimately related at the molecular level (Bernoux et al., submitted). This work suggests the potential existence of relatively simple (in a genetic sense) functional constraints associated with the breadth of pathogen elicitors that are recognized by resistance proteins.

## Conclusions

We conclude that network approaches offer great utility as a tool for probing the ecological and evolutionary genetics of host-pathogen interactions. Prior analyses of the datasets used in this study did not attempt to estimate nor dissect the network characteristics examined here, and the network meta-analysis has revealed several novel results that complement and extend findings revealed in previous studies.

First, we found that host resistance (and by extension pathogen infectivity) strategies are consistently variable within and among networks, corresponding to a spectrum of specialists and generalists being maintained within all networks. While the maintenance of diversity for resistance and infectivity is well known in this system, the network approaches we have utilized reveal novel information regarding the distribution and statistical structure of these specificities. Relationships between patterns of specialization and partial resistance further suggest evolutionary trade-offs between specialization and the strength of resistance. Second, we found that all networks were significantly nested. While we hypothesized that temporal evolutionary dynamics might be important for the development of nestedness in host-pathogen infection networks, there was little evidence to suggest that this was so. Rather, the common patterns observed in terms of nestedness suggest the existence of general determinants across networks (e.g., trade-offs or underlying genetics). Third, we found that resistance networks were significantly modular in two spatial networks, clearly reflecting spatial and ecological dynamics within one of the networks, and perhaps reflecting genetic structure within networks more generally. Together, these results demonstrate that analysis of the topology of bipartite interaction networks has the potential to provide important information regarding the genetic interactions that underpin disease outcomes and coevolutionary dynamics within host-pathogen associations. In particular, our results demonstrate that network approaches have potential to complement more commonly used approaches for analysing population and temporal-based sampling by placing a more explicit focus on individual variation in patterns of specificity, and revealing structure (i.e., nestedness and modularity) in the data that are independent of common *a priori* hypotheses (e.g., local adaption).

Finally, in terms of new directions, we suggest that network approaches may offer great utility for dissecting the genetic nature of host-pathogen interactions from population level data, perhaps in combination with other approaches (Heath and Nuismer, 2014). In particular, we suggest that in systems where performing classical genetics is problematic, network structure has potential to reveal the genetic architecture underlying antagonistic host-pathogen interactions (e.g., GFG, MA). In particular, these genetic models are predicted to generate contrasting patterns in terms of modularity (high for MA) and nestedness (high for GFG) (Moury et al., 2014). We suggest that a useful goal for future theoretical studies is to examine the role of life-history (e.g., dispersal) and contrasting genetics in driving patterns of nestedness and modularity in host-pathogen networks.

### Conflict of interest statement

The authors declare that the research was conducted in the absence of any commercial or financial relationships that could be construed as a potential conflict of interest.
